# GDP vs. LDP: A Survey from the Perspective of Information-Theoretic Channel

**DOI:** 10.3390/e24030430

**Published:** 2022-03-19

**Authors:** Hai Liu, Changgen Peng, Youliang Tian, Shigong Long, Feng Tian, Zhenqiang Wu

**Affiliations:** 1 Guizhou Big Data Academy, Guizhou University, Guiyang 550025, China; liuhai@snnu.edu.cn; 2 College of Computer Science and Technology, Guizhou University, Guiyang 550025, China; yltian@gzu.edu.cn (Y.T.); sglong@gzu.edu.cn (S.L.); 3 State Key Laboratory of Public Big Data, Guizhou University, Guiyang 550025, China; 4 School of Computer Science, Shaanxi Normal University, Xi’an 710119, China; tianfeng@snnu.edu.cn (F.T.); zqiangwu@snnu.edu.cn (Z.W.)

**Keywords:** GDP vs. LDP, information-theoretic channel, Rényi divergence, mutual information, expected distortion

## Abstract

The existing work has conducted in-depth research and analysis on global differential privacy (GDP) and local differential privacy (LDP) based on information theory. However, the data privacy preserving community does not systematically review and analyze GDP and LDP based on the information-theoretic channel model. To this end, we systematically reviewed GDP and LDP from the perspective of the information-theoretic channel in this survey. First, we presented the privacy threat model under information-theoretic channel. Second, we described and compared the information-theoretic channel models of GDP and LDP. Third, we summarized and analyzed definitions, privacy-utility metrics, properties, and mechanisms of GDP and LDP under their channel models. Finally, we discussed the open problems of GDP and LDP based on different types of information-theoretic channel models according to the above systematic review. Our main contribution provides a systematic survey of channel models, definitions, privacy-utility metrics, properties, and mechanisms for GDP and LDP from the perspective of information-theoretic channel and surveys the differential privacy synthetic data generation application using generative adversarial network and federated learning, respectively. Our work is helpful for systematically understanding the privacy threat model, definitions, privacy-utility metrics, properties, and mechanisms of GDP and LDP from the perspective of information-theoretic channel and promotes in-depth research and analysis of GDP and LDP based on different types of information-theoretic channel models.

## 1. Introduction

It is assumed that an attacker has background knowledge of name information about *n* patients in a medical dataset with a certain disease. The attacker can statistically query the sum of disease status of n−1 patients except the *i*-th patient and the sum of disease status with all *n* patients and then can infer whether the *i*-th patient has a disease by comparing the two statistical query results. To mitigate the problem of individual privacy leakage caused by the above statistical inference attack, Dwork et al. [1] proposed differential privacy (DP) to protect individual privacy independent of the presence or absence of any individual. Since DP requires that the data collector is trustworthy in a centralized setting, it is called centralized DP. Moreover, because DP considers global sensitivity of adjacent datasets, it is also known as global differential privacy (GDP). However, the data collector is untrusted in real-world applications. Therefore, Kasiviswanathan et al. [2] proposed that local differential privacy (LDP) allows an untrusted third party to perform statistical analysis while achieving user’s privacy by random perturbation of local data. Both GDP and LDP have privacy-utility monotonicity and can achieve privacy-utility tradeoff [3]. GDP and LDP have become popular methods of data privacy preserving of the centralized and local setting, respectively. However, GDP and LDP have different advantages and disadvantages. In Table 1, we agree with Dobrota’s [4] comparative analysis results of the advantages and disadvantages of GDP and LDP.

Because of the advantages of using GDP and LDP in the centralized and local setting, respectively, the data privacy community has widely studied GDP and LDP based on information theory. The current work focuses on GDP and LDP from the following aspects based on information theory, including privacy threat model, channel models and definitions of GDP and LDP, privacy-utility metrics of GDP and LDP, properties of GDP and LDP, and mechanisms satisfying GDP and LDP. Unless otherwise stated, the information-theoretic channel model refers to the discrete single symbol information-theoretic channel in this survey. However, there is no review work to systematically survey the above existing work on GDP and LDP from the perspective of information-theoretic channel.

Therefore, this paper systematically surveyed GDP and LDP under the information-theoretic channel model from the aspects of resisting privacy threat model, channel models, definitions, privacy-utility metrics, properties, and achieving mechanisms. Our main contributions are as follows.

(1) We summarized the privacy threat model under information-theoretic channel, and we provided a systematic survey on channel models, definitions, privacy-utility metrics, properties, and mechanisms of GDP and LDP from the perspective of information-theoretic channel.

(2) We presented a comparative analysis between GDP and LDP from the perspective of information-theoretic channel. Then, we concluded the common channel models, definitions, privacy-utility metrics, properties, and achieving mechanisms of GDP and LDP in the existing work.

(3) We surveyed applications of GDP and LDP in synthetic data generation. Specifically, we first presented the membership inference attack and model extraction attack against generative adversarial network (GAN). Then, we reviewed the differential privacy synthetic data generation with GAN and differential privacy synthetic data generation with federated learning, respectively.

(4) Through analyzing the advantages and disadvantages of the existing work for different application scenarios and data types, we also discussed the open problems of GDP and LDP based on different types of information-theoretic channel models in the future.

This paper is organized as follows. Section 2 introduces the preliminaries. Section 3 summarizes the privacy threat model of centralized and local data setting under information-theoretic channel. Section 4 describes the channel models of GDP and LDP and uniformly states and analyzes the definitions of GDP and LDP under their channel models. Section 5 summarizes and compares the information-theoretic privacy-utility metrics of GDP and LDP. In Section 6, we present and analyze the properties of GDP and LDP from the perspective of information-theoretic channel. Section 7 summarizes and analyzes the mechanisms of GDP and LDP from the perspective of information-theoretic channel. Section 8 discusses the open problems of GDP and LDP from the perspective of different types of information-theoretic channel on different application scenarios and data types. Section 9 concludes this paper.

## 2. Preliminaries

In this section, we introduce the preliminaries of GDP [1], LDP [5], and the information-theoretic channel model and metrics [5,6,7,8,9,10,11]. The commonly used mathematical symbols are summarized in Table 2.

### 2.1. GDP and LDP

A dataset *x* is collections of records coming from a universal set *X*, and each $x_{i}$ denotes the *i*-th item or a subset in the dataset *x*. When two datasets are different in only one item, the two datasets are adjacent datasets.

**Definition** **1** (GDP)**.** *A randomized mechanism M with domain X is (ε,δ)-DP if for all S⊆Range(M) and for any two adjacent datasets $x,x^{\prime }\in X$, it holds*

(1) $$p\left(M\left(x\right)\in S\right)\leq e^{\varepsilon }p\left(M\left(x^{\prime }\right)\in S\right)+\delta$$

*where the probability space is over the coin flips of the mechanism M. If δ=0, then M is ε-DP.*

The coin flips of the mechanism M mean that a DP mechanism M inherently has only equally likely outcomes with regard to each record of each individual. The equally likely to occur means that the probability distribution of response to any query is the same independent of any individual opting presence or absence in the dataset. If M is (ε,δ)-DP, then M is ε-DP with probability at least 1−δ for all datasets *x* and $x^{\prime }$ when *x* and $x^{\prime }$ are adjacent datasets. For the definition of LDP, the coin flips of mechanism M have the same meanings.

**Definition** **2** (LDP)**.** *A randomized mechanism M satisfies ε-LDP if and only if for any pairs input values x and $x^{\prime }$ in the domain of X, and for any possible output z∈Range(M), it holds*

(2) $$p\left(M\left(z|x\right)\right)\leq e^{\varepsilon }p\left(M\left(z|x^{\prime }\right)\right)+\delta$$

*where the probability space is over the coin flips of the mechanism M. If δ=0, then M is ε-LDP.*

### 2.2. Information-Theoretic Channel and Metrics

The mathematical model of an information-theoretic channel can be denoted by (X,p(y|x),Y), where

(1) *X* is an input random variable, and its value set is $x=\{x_{1},x_{2},\ldots ,x_{n}\}$.

(2) *Y* is an output random variable, and its value set is $y=\{y_{1},y_{2},\ldots ,y_{m}\}$.

(3) p(y|x) is the channel transition probability matrix, and the sum of the probabilities in each row satisfies $\sum_{j=1}^{m}p\left(y_{j}|x_{i}\right)=1$.

In information-theoretic channel model, the Rényi divergence of a probability distribution $p\left(x\right)=(p\left(x_{1}\right),p\left(x_{2}\right),\ldots ,p\left(x_{n}\right))$ on source *X* from another distribution $q\left(x\right)=(q\left(x_{1}\right),q\left(x_{2}\right),\ldots ,q\left(x_{n}\right))$ is $D_{\alpha }\left(p\left(x\right)||q\left(x\right)\right)=\frac{1}{\alpha -1}\log_{2}\sum_{i=1}^{n}{\left(p\left(x_{i}\right)\right)}^{\alpha }{\left(q\left(x_{i}\right)\right)}^{1-\alpha }$, where α>0 and α≠1. When q(x) is the uniform distribution with $q\left(x\right)=\left(\frac{1}{n},\ldots ,\frac{1}{n}\right)$, the Rényi entropy is $H_{\alpha }\left(X\right)=\frac{1}{1-\alpha }\log_{2}\left(\sum_{i=1}^{n}p{\left(x_{i}\right)}^{\alpha }\right)$ in terms of the Rényi divergence of p(x). When α→1, the Rényi entropy tends to the Shannon entropy $H\left(X\right)=\lim_{\alpha \to 1}H_{\alpha }\left(X\right)=-\sum_{i=1}^{n}p\left(x_{i}\right)\log_{2}p\left(x_{i}\right)$ of source *X*. When α→∞, the Rényi entropy tends to the min-entropy $H_{\infty }\left(X\right)=\lim_{\alpha \to \infty }H_{\alpha }\left(X\right)=-\log_{2}\max_{x_{i}\in X}p\left(x_{i}\right)$. The conditional Rényi entropy of *X* given *Y* is $H_{\alpha }\left(X|Y\right)=-\log_{2}{\left(\sum_{j=1}^{m}p\left(y_{j}\right){\left(\sum_{i=1}^{n}{\left(p\left(x_{i}|y_{j}\right)\right)}^{\alpha }\right)}^{\frac{1}{\alpha }}\right)}^{\frac{\alpha }{\alpha -1}}$. When α→1, the conditional Rényi entropy is conditional Shannon entropy $H\left(X|Y\right)=\lim_{\alpha \to 1}H_{\alpha }\left(X|Y\right)=\sum_{i=1}^{n}\sum_{j=1}^{m}p\left(x_{i}y_{j}\right)\log_{2}p\left(x_{i}|y_{j}\right)$. When α→∞, the conditional Rényi entropy is conditional min-entropy $H_{\infty }\left(X|Y\right)=\lim_{\alpha \to \infty }H_{\alpha }\left(X|Y\right)=-\log_{2}\sum_{j=1}^{m}p\left(y_{j}\right)\max_{x_{i}\in X}p\left(x_{i}|y_{j}\right)$. The mutual information I(X;Y)=H(X)−H(X|Y) is the average information measure of *X* contained in random variable *Y*. Furthermore, the max-information is $I_{\infty }\left(X;Y\right)=H_{\infty }\left(X\right)-H_{\infty }\left(X|Y\right)=\max\log_{2}\frac{p(x_{i},y_{j})}{p\left(x_{i}\right)p\left(y_{j}\right)}$, and the β-approximate max-information is $I_{\infty }^{\beta }\left(X;Y\right)=\max\log_{2}\frac{p(x_{i},y_{j})-\beta }{p\left(x_{i}\right)p\left(y_{j}\right)}$.

Moreover, when α→1, the Rényi divergence is Kullback–Leibler (KL) divergence $D_{\mathrm{KL}}\left(p\left(x\right)||q\left(x\right)\right)=\lim_{\alpha \to 1}D_{\alpha }\left(p\left(x\right)||q\left(x\right)\right)=\sum_{i=1}^{n}p\left(x_{i}\right)\log_{2}\frac{p(x_{i})}{q(x_{i})}$. The KL-divergence is an instance of the family of *f*-divergence $\Delta_{f}\left(p\left(x\right),q\left(x\right)\right)=\sum_{i=1}^{n}q\left(x_{i}\right)f\left(\frac{p(x_{i})}{q(x_{i})}\right)$ with non-negative convex functions f(t)=tlnt−t+1. The total variation distance is also an instance of the family of *f*-divergence with $f\left(t\right)=\frac{1}{2}\left|t-1\right|$, and the total variation distance between distributions p(x) and q(x) is $||p\left(x\right)-{q\left(x\right)||}_{TV}=\frac{1}{2}||p\left(x\right)-{q\left(x\right)||}_{1}$. When α→∞, the Rényi divergence is is max-divergence $D_{\infty }\left(p\left(x\right)||q\left(x\right)\right)=\max_{x_{i}\in X}\log_{2}\frac{p(x_{i})}{q(x_{i})}$, and the δ-approximate max-divergence is $D_{\infty }^{\delta }\left(p\left(x\right)||q\left(x\right)\right)=\max_{x_{i}\in X}\log_{2}\frac{p(x_{i})-\delta }{q(x_{i})}$.

The expected distortion between input random variable *X* and output random variable *Y* is

(3) $$\bar{D}=\sum_{i=1}^{n}\sum_{j=1}^{m}p\left(x_{i}y_{j}\right)d\left(x_{i},y_{j}\right)=\sum_{i=1}^{n}\sum_{j=1}^{m}p\left(x_{i}\right)p\left(y_{j}|x_{i}\right)d\left(x_{i},y_{j}\right)$$

where the distance measurement $d(x_{i},y_{j})$ is single symbol distortion. The average error probability is

(4) $$p_{E}=\sum_{i=1}^{n}p\left(x_{i}\right)\sum_{j=1}^{m}p\left(y_{j}\neq x_{i}|x_{i}\right)$$

Thus, the average error probability is expected Hamming distortion, when $d(x_{i},y_{j})$ is Hamming distortion in Equation (3).

## 3. Privacy Threat Model on Information-Theoretic Channel

To mitigate the statistical inference attack, the GDP has a strong adversary assumption in which an adversary knows n−1 dataset records and tries to identify the remaining one [12,13]. However, the adversary is usually computationally bounded. Thus, Mironov [11] and Mir [14] assumed that the adversary has prior knowledge over the set of possible input dataset *X*. Furthermore, Smith [15] proposed one-try attack, where an adversary is allowed to ask exactly one question about form, “is $X=x_{i}$?”. The Rényi min-entropy of *X* denotes the probability of success for one-try attack with the best strategy, which chooses the $x_{i}$ with maximum probability. The conditional Rényi min-entropy of *X* given *Y* captures the probability of guessing the value of *X* in one single try when the output of *Y* is known. Therefore, the privacy leakage of channel model is Rényi min-entropy leakage $I_{\infty }\left(X;Y\right)=H_{\infty }\left(X\right)-H_{\infty }\left(X|Y\right)$ under one-try attack [7]. The Rényi min-entropy leakage is max-information, and it is the ratio of the probabilities of attack success with a priori probability and a posterior probability. Thus, a Rényi min-entropy leakage corresponds to the concept of Bayes risk, which can also be regarded as a measure of the effectiveness of the attack. The maximal leakage $\max_{p(x)}I_{\infty }\left(X;Y\right)$ is the maximal reduction in uncertainty about *X* when *Y* is observed [16]. The maximal leakage is taken by maximizing over all input distributions.

When adversary possesses knowledge of a priori probability distribution of input, LDP can lead to the risk of privacy leakage [2,17,18,19,20,21,22]. However, a better privacy-utility tradeoff can be achieved by incorporating the attacker’s knowledge into the LDP. Therefore, data utility can be improved by explicitly modeling the adversary’s prior knowledge of the LDP.

To sum up, the privacy threat of information-theoretic channel refers to the Bayes risk on input *X*, when attack known output *Y*. Thus, GDP and LDP can be used to mitigate the above privacy threat on information-theoretic channel for numerical data and categorical data, respectively.

## 4. Information-Theoretic Channel Models and Definitions of GDP and LDP

In this section, we summarize and compare information-theoretic channel models of GDP and LDP. Furthermore, we present the information-theoretic definitions of GDP and LDP under their information-theoretic channel models and compare the definitions of GDP (LDP) with other information-theoretic privacy definitions.

### 4.1. Information-Theoretic Channel Models of GDP and LDP

In Table 3, Alvim et al. [7] had constructed an information-theoretic channel model (X,P(z|x),Z) of GDP to any query function f:X→Y of adjacent datasets, where P(z|x) is DP mapping on input dataset *X* and random output *Z* of real output *Y*. Similarly, we can also construct an information-theoretic channel model (X,p(z|x),Z) of LDP to any different single input *x* and $x^{\prime }$, where p(z|x) is LDP mapping on categorical dataset x={0,1,…,n−1} of single input and categorical dataset z={0,1,…,n−1} of single random output. Next, we will survey and compare the information-theoretic definitions of GDP and LDP under the above given information-theoretic channel models.

### 4.2. Information-Theoretic Definitions of GDP and LDP

In Table 4, we summarize the current work on definitions of GDP using different information-theoretic metrics under the information-theoretic channel model. Alvim et al. [7] intuitively gave the definition of ε-DP using transition probability distribution p(z|x) for all z∈Z, $x,x^{\prime }\in X$ with adjacent datasets *x* and $x^{\prime }$. Barthe and Olmedo [8] defined (ε,δ)-DP based on *f*-divergence, which is a redefinition of DP. Dwork and Roth [9] gave the definitions of ε-DP and (ε,δ)-DP based on max-divergence, which is an equivalent definition of DP from the perspective of information-theoretic channel. Mironov [11] defined the (α,ε)-Rényi DP (RDP) using Rényi divergence, and (α,ε)-RDP satisfies $\left(\varepsilon +\frac{\log\frac{1}{\delta }}{\alpha -1},\delta \right)$-DP. When α→∞, (α,ε)-RDP is ε-DP according to the max-divergence. Conversely, ε-DP is $\left(\alpha ,\frac{1}{2}\varepsilon^{2}\alpha \right)$-RDP [23]. We can conclude that RDP is a generalization of GDP. When α→1, (1,ε)-RDP is the definitions of (ε,δ)-DP based on the KL-divergence of Reference [8]. When α→∞, (∞,ε)-RDP is the definitions of ε-DP and (ε,δ)-DP based on the maximum divergence of Reference [9]. According to the *f*-divergence, Asoodeh et al. [24] also established the optimal relationship between RDP and (ε,δ)-DP to help to derive the optimal (ε,δ)-DP parameters of a mechanism for a given level of RDP. Chaudhuri et al. [25] defined (H,Γ)-capacity bounded DP based on H-restricted divergence, where H is a class of functions and Γ is a divergence. The (H,Γ)-capacity bounded DP relaxes GDP by restricting the adversary to attack or post-process the output of a privacy mechanism using functions drawn from a restricted function class H and models adversaries of this form with restricted *f*-divergences between probability distributions on datasets different from a single record. The H-restricted *f*-divergence is $D_{f}^{H}\left(p\left(x\right),q\left(x\right)\right)=\sup_{h\in H}E_{x∼p(x)}\left[h\left(x\right)\right]-E_{x∼q(x)}\left[f^{*}\left(h\left(x\right)\right)\right]$, where $f^{*}$ is Fenchel conjugate and $f^{*}\left(s\right)=\sup_{x\in R}x\cdot \left(s-f\left(x\right)\right)$. The H-restricted Rényi divergence is $D_{R,\alpha }^{H}\left(p\left(x\right),q\left(x\right)\right)=\frac{\log(1+\alpha \left(\alpha -1\right)D_{\alpha }^{H}\left(p\left(x\right),q\left(x\right)\right))}{\alpha -1}$, where $D_{\alpha }^{H}$ is the H-restricted Rényi divergence of order α. When H is the class of all functions and Γ is the Rényi divergence, this definition reduced to RDP. Additionally, when Γ is the *f*-divergence, this definition is (ε,δ)-DP of Reference [8]. Thus, capacity bounded DP is a generalization of RDP.

We compare the other information-theoretic privacy definitions and GDP under the information-theoretic channel model in Table 5. Calmon and Fawaz [26] provided ε-information privacy, which is stronger than 2ε-DP. Makhdoumi and Fawaz [27] also showed that ε-information privacy is much stronger than 2ε-DP, ε-strong DP is stronger than ε-information privacy, and ε-DP is stronger than (ε,δ)-DP. Wang et al. [12] analyzed the relation between identifiability, DP, and mutual-information privacy and demonstrated that ε-identifiability is stronger than $\left[\varepsilon -\max\ln\frac{p(x)}{p(x^{\prime })},\varepsilon \right]$-DP and ε-DP is stronger than $\left[\varepsilon ,\varepsilon +2\max\ln\frac{p(x)}{p(x^{\prime })}\right]$-mutual-information privacy. Cuff and Yu [13] also proved that ε-DP is stronger than ε-mutual-information DP and ε-mutual-information DP is stronger than (ε,δ)-DP, where ε-mutual-information DP is ε-mutual-information privacy of Reference [12].

In the information-theoretic channel model of LDP of Table 3, we use the convex polytope proposed by Holohan et al. [28] as the general definition of the LDP. Thus, the definition of LDP for any different single input *x* and $x^{\prime }$ and Hamming distance $\Delta (x,x^{\prime })=1$ is

(5) $$\left\{p\left(z|x\right):e^{\varepsilon }=\max\left\{\frac{p(z|x)}{p(z|x^{\prime })}\right\}\right\}$$

where $\sum_{z}p\left(z|x\right)=1$ and p(z|x)≥0.

In Table 6, we make the comparative analysis of other information-theoretic privacy definitions and LDP under information-theoretic channel model. Jiang et al. [19] compared LDP, mutual-information privacy [12], and local information privacy, where local information privacy is information privacy of Reference [26]. When privacy budget is ε, ε-local information privacy is stronger than ε-mutual-information privacy and 2ε-LDP, and ε-LDP is stronger than ε-local information privacy. Lopuhaä-Zwakenberg et al. [21] also showed the same conclusion above and also proved that ε-side-channel resistant local information privacy (SRLIP) is stronger than ε-local information privacy when the privacy budget is ε.

## 5. Privacy-Utility Metrics of GDP and LDP under Information-Theoretic Channel Models

In Table 7, we summarize and analyze the information-theoretic privacy metrics of GDP. When α→∞, Rényi divergence is used as the privacy metric of GDP, which is a natural relaxation of GDP based on the Rényi divergence [11]. Chaudhuri et al. [25] used restricted divergences $D_{\Gamma }^{H}\left(p\left(z|x\right),p\left(z|x^{\prime }\right)\right)$ as privacy metric. When Γ is Rényi divergence, the capacity bounded DP is a generalization of RDP. When Γ is *f*-divergence, the capacity bounded DP is (ε,δ)-DP in [8]. In [14,29], mutual information is used as the privacy metric of GDP, which is the amount of information leaked on *X* after observing *Z*. Cuff and Yu [13] also used α-mutual-information as the privacy metric of GDP, which is the generalization of mutual information using Rényi divergence of order α. Alvim et al. [7] used min-entropy leakage as the privacy metric of GDP, which is the ratio of the probabilities of right guessing a priori and a posterior. Furthermore, maximal leakage of channel p(z|x) is used as the privacy metric of GDP, which is the maximal reduction in uncertainty of *X* when *Z* is given [7,16]. According to the graph symmetrization, Edwards et al. [30] also regarded min-entropy leakage as an important measure of differential privacy loss of information channels under Blowfish privacy. Blowfish privacy is a generalization of global differential privacy. Rogers et al. [31] defined the privacy metric of GDP using max-information and β-approximate max-information, which are a correlation measure allowing to bound the change in the conditional probability of an event relative to the prior probability. In [32,33], the privacy budget is directly used as privacy metric. Therefore, we can conclude that Rényi divergence is a more general privacy metric of GDP, since Rényi divergence is a generalization of restricted divergences and it can deduce *f*-divergence, min-entropy leakage, maximal leakage, and max-information. Moreover, mutual information can also be used as a privacy metric of GDP.

We also summarize and analyze the information-theoretic utility metrics of GDP in Table 8. In the information-theoretic channel model of GDP, expected distortion is mainly the utility measurement method, which shows how much information about the real answer can be obtained from the reported answer to average [7,33]. Padakandla et al. [32] used fidelity as the utility metric, and the fidelity between transition probability distributions is measured by $L_{1}$-distortion metric. Mutual information is not only used as a privacy metric but also as a utility metric of GDP, which captures the amount of information shared by two variables [33].

In Table 9, we summarize and analyze existing work of information-theoretic privacy metrics of LDP. In the information-theoretic channel model of LDP, Duchi et al. [17] defined the privacy metric of LDP using KL-divergence, which bounds the KL-divergence between distributions p(z|x) and $p(z|x^{\prime })$ by a quantity dependent on the privacy budget ε and gives the upper bound of KL-divergence by combining with the total variation distance between $p_{1}\left(x_{i}\right)$ and $p_{2}\left(x_{i}\right)$ of the initial distributions of the $x_{i}$. Of course, mutual information can also be used as a privacy measure of LDP [34,35]. More generally, the existing work mainly uses the definition of the LDP as the privacy metric [5,36,37,38]. In [39], Lopuhaä-Zwakenberg et al. gave an average privacy metric based on the ratio of conditional entropy of sensitive information *X*.

Next, we summarize and analyze the information-theoretic utility metric of LDP in Table 10. In the information-theoretic channel model of LDP, *f*-divergence [5] and mutual information [5,36,38] can also be used as utility measures of LDP. In most cases, expected distortion is used as the utility measure of LDP [20,34,35,36,37]. In [39], Lopuhaä-Zwakenberg et al. presented distribution utility and tally utility metrics based on the ratio of relevant information.

## 6. Properties of GDP and LDP under Information-Theoretic Channel Models

In Table 11, we present and analyze the properties of GDP based on the information-theoretic channel model. According to the Rényi divergence, Mironov [11] demonstrated that the new definition shares many important properties with the standard definition of GDP, including post-processing, group privacy, and sequential composition. Considering H-restricted divergences including Rényi divergence, Chaudhuri et al. [25] showed that capacity bounded DP has properties of post-processing, convexity, sequential composition, and parallel composition. Barthe and Köpf [16] proved the sequential composition and parallel composition of GDP based on maximal leakage under the information-theoretic channel model. Barthe and Olmedo [8] also proved the parallel composition of GDP using *f*-divergence. We know that Rényi divergence can deduce maximal leakage and max-divergence. *f*-divergence of Reference [8] is actually max-divergence. Thus, we can conclude that, such as post-processing, convexity, group privacy, and sequential composition, and parallel composition, the properties of GDP can be proved by using Rényi divergence.

Similarly, GDP and LDP share the above properties under the information-theoretic channel model. Therefore, LDP also has the properties of post-processing, convexity, group privacy, and sequential composition, and parallel composition.

Moreover, we have showed that GDP and LDP have privacy-utility monotonicity [3]. In GDP, (ε,δ)-DP shows

(6) $$\frac{p(z|x)-\delta }{p(z)}=\frac{p(z|x)-\delta }{\sum_{x^{\prime }}p\left(z|x^{\prime }\right)p\left(x^{\prime }\right)}\leq \frac{p(z|x)-\delta }{\sum_{x^{\prime }}\left(p\left(z|x\right)-\delta \right)p\left(x^{\prime }\right)e^{-\varepsilon }}=e^{\varepsilon }$$

We can obtain

(7) $$\sum_{x}\sum_{z}p\left(xz\right)\log\frac{p(z|x)-\delta }{p(z)}\leq \varepsilon \leq I\left(X;Z\right)$$

When δ=0, we have

(8) $$I\left(X;Z\right)=\sum_{x}\sum_{z}p\left(xz\right)\log\frac{p(z|x)}{p(z)}\leq \varepsilon$$

We can obtain I(X;Z)=ε. We use mutual information as the utility metric. We can conclude that the mutual information of GDP decreases as the decreasing of the privacy budget, and vice versa. Privacy preserving is stronger and the utility is worse, and vice versa. Thus, GDP has privacy-utility monotonicity indicating the privacy-utility tradeoff. Similarly, we can observe that LDP also has privacy-utility monotonicity indicating the privacy-utility tradeoff.

## 7. GDP and LDP Mechanisms under Information-Theoretic Channel Models

In Table 12, we summarize and compare the GDP mechanisms from the perspective of information-theoretic channel on uniform distribution of the source *X*. Alvim et al. [7] maximized expected distortion under min-entropy leakage constraint and obtained the optimal randomization mechanism using graph symmetry caused by the adjacent relationship between adjacent datasets. The optimal randomization mechanism can ensure better utility while achieving ε-DP. According to the risk-distortion framework, Mir [14] minimized mutual information when the constraint condition is expected distortion and obtain ε-DP mechanism $p\left(z|x\right)=\frac{p(z)\exp(-\varepsilon d(x,z))}{Z(x,\varepsilon )}$ by Lagrangian multipliers method, where Z(x,ε) is a normalization function. GDP mechanism of [14] is corresponding to the exponential mechanism [40]. The conditional probability distribution p(z|x) minimizes the privacy leakage risk given a distortion constraint. Ayed et al. [33] maximized mutual information when constraint condition is DP and solved the constrained maximization program to obtain DP mapping under strongly symmetric channel.

In addition, Mironov [11] analyzed the RDP of three basic mechanisms and their self-composition, including randomized response, Laplace mechanism, and Gaussian mechanism, and gave the parameters of RDP of these mechanisms. Considering a linear adversary and unrestricted adversary, Chaudhuri et al. [25] also discussed the capacity bounded DP properties of Laplace mechanism, Gaussian mechanism, and matrix mechanism and presented the bound of privacy budget ε of Laplace mechanism and Gaussian mechanism under KL-divergence and Rényi divergence, respectively.

In Table 13, we summarize and compare the LDP mechanisms from the perspective of information-theoretic channel under uniform distribution of the source *X*. According to the rate-distortion function, References [34,35,37] maximized mutual information under expected Hamming distortion *D* constraint and obtained privacy budget $\varepsilon =\log\frac{1-D}{D}$ for binary channel and privacy budget $\varepsilon =\log\left(m-1\right)+\frac{1-D}{D}$ for discrete alphabets. Kairouz et al. [5] maximized KL-divergence and mutual information under LDP constraint and obtained binary randomized response mechanism, multivariate randomized response mechanism, and quaternary randomized response mechanism by solving the privacy-utility maximization problem, which is equivalent to solving the finite-dimensional linear program. Although Ayed et al. [33] maximized mutual information about GDP constraint, they also obtained binary randomized response mechanism and multivariate randomized response mechanism under a strongly symmetric channel. Wang et al. [38] maximized mutual information on LDP constraint and obtained the *k*-subset mechanism with respect to the uniform distribution on the source *X*. When k=1, the 1-subset mechanism is the multivariate randomized response mechanism. When n=2 and k=1, the multivariate randomized response mechanism is the binary randomized response mechanism. Xiong et al. [36] minimized privacy budget $\varepsilon =\max_{x,x^{\prime },z}\frac{p(z|x)}{p(z|x^{\prime })}$ under expected distortion constraint, which is equivalent to solving a standard generalized linear-fractional program via the bisection method. However, Xiong et al. [36] did not give a specific expression of the optimal privacy channel p(z|x).

Furthermore, Duchi et al. [41] showed that randomized response is an optimal way to perform survey sampling while maintaining privacy of the respondents. Holohan et al. [42] proposed following optimal mechanism of randomized response satisfying (ε,δ)-DP under uniform distribution of the source *X*, which is

(9) $$p\left(z|x\right)=\left\{\begin{array}{cc} \frac{e^{\varepsilon }+\delta }{1+e^{\varepsilon }}, & x=z\\ \frac{1-\delta }{1+e^{\varepsilon }}, & x\neq z \end{array}\right.$$

Erlingsson et al. [43] proposed randomized aggregatable privacy-preserving ordinal response (RAPPOR) by applying randomized response in a novel manner. RAPPOR provides privacy guarantee using permanent randomized response and instantaneous randomized response and ensures high-utility analysis of the collected data. RAPPOR encodes each value υ into a length-*k* binary bit vector *B*. For permanent randomized response, RAPPOR generates $B_{1}$ with the probability

(10) $$p\left(B_{1}\left[\upsilon \right]=1\right)=\left\{\begin{array}{cc} 1-\frac{1}{2}f, & B[\upsilon ]=1\\ \frac{1}{2}f, & B[\upsilon ]=0 \end{array}\right.$$

where $f=\frac{2}{e^{\frac{\varepsilon }{2}}+1}$. With respect to instantaneous randomized response, RAPPOR perturbs $B_{1}$ with the probability

(11) $$p\left(B^{*}\left[i\right]=1\right)=\left\{\begin{array}{cc} p, & B_{1}\left[i\right]=1\\ q, & B_{1}\left[i\right]=0 \end{array}\right.$$

## 8. Differential Privacy Synthetic Data Generation

Data sharing facilitates training better models, decision making, and the reproducibility of scientific research. However, if the data are shared directly, it will face the risk of privacy leakage and the problem of small training sample size. Thus, synthetic data are often used to replace the sharing of real data. At present, one of the main methods for synthetic data generation is generative adversarial network [44]. GAN consists of two neural networks: one is a generator, and the other is a discriminator. The generator generates a realistic sample by inputting a noise obeying multivariable Gaussian distribution or uniform distribution. The discriminator is a binary classifier (such as 0–1 classifier) to judge whether the input sample is real or fake. In other words, the discriminator can distinguish whether each input sample comes from the real sample set or the fake sample set. However, the generator makes the ability of making samples as strong as possible so that the discriminator cannot judge whether the input sample is a real sample or a fake sample. According to this process, GAN can generate synthetic data to approximate the real data. Because the synthetic data accurately reflect the distribution of training data, it can avert privacy leakage by replacing real data sharing, augment small-scale training data, and be generated as desired. Thus, GAN can generate synthetic data for time series, continuous, and discrete data [45].

However, because the discriminator easily memorizes the training data, it brings the risk of privacy leakage [46]. Therefore, GAN mainly faces the privacy threat of membership inference attack and model extraction attack in Table 14. Hayes et al. [47] proposed a membership inference attack against the generative models, which means that the attacker can determine whether it is used to train the model given a data point. Liu et al. [48] proposed a new membership inference attack, co-membership inference attack, which checks whether the given *n* instances are in the training data, where the prior knowledge is completely used or not at all in the training. Hilprecht et al. [49] proposed a Monte Carlo attack on the membership inference against generative models, which yields high membership inference accuracy. Chen et al. [50] systematically analyzed the potential risk of privacy leakage caused by the generative models and proposed the classification of membership inference attacks, including not only the existing attacks but also the proposed generic attack model based on reconstruction. Hu and Pang [51] studied the model extraction attack against GAN by stealing the machine learning model whose purpose is to copy the machine learning model through query access to the target model. In order to mitigate the model extraction attack, Hu and Pang designed defenses based on input and output perturbation by perturbing latent code and generating samples, respectively.

However, the existing work mainly achieves the model protection of neural network based on differential privacy. By using the $\ell_{2}$ norm of the gradient and the clipping threshold to clip the gradient, and using the Gaussian mechanism to randomly perturb the clipping gradient, Abadi et al. [52] proposed differential privacy stochastic gradient decent (DP-SGD) to protect the privacy of training data during the training process and demonstrated the moment accountant of the privacy loss that provides a tighter bound on the privacy loss compared to the generic strong composition theorem of differential privacy [9].

Next, in Table 15 and Table 16, we mainly review the work of synthetic data generation based on differential privacy GAN and differential privacy GAN with federated learning from the following aspects: gradient perturbation, weight perturbation, data perturbation, label perturbation, and objective function perturbation. Thus, our work is different from the existing surveys [53,54].

### 8.1. Differential Privacy Synthetic Data Generation with Generative Adversarial Network

Because the discriminator of GAN can easily remember the training samples, training GAN with sensitive or private data samples breaches the privacy of the training data. Thus, using gradient perturbation can protect the privacy of the sensitive training data by training GAN models with differential privacy based on DP-SGD. Existing work protects the privacy of the training dataset by adding carefully designed noise to clipping gradients during the learning procedure of discriminator and uses moment accountant or RDP accountant to better keep track of the privacy cost for improving the quality of synthetic data. RDP accountant [11] provides a tighter bound for privacy loss in comparison with the moment accountant. In gradient perturbation, clipping strategy and perturbation strategy improve the performance of the model while preserving privacy of the training dataset.

Using gradient perturbation, Lu and Yu [55] proposed a unified framework for publishing differential privacy data based on GAN, such as tabular data and graphs, and synthetic data with acceptable utility in differential privacy manner. Xie et al. [56] proposed a differential privacy Wasserstein GAN (WGAN) [57] model, which adds carefully designed noise to the clipping gradient in the learning process, generates high-quality data points at a reasonable privacy level, and uses moment accountant to ensure the privacy in the iterative gradient descent process. Frigerio et al. [45] developed a differential privacy framework for privacy protection data publishing using GAN, which can easily adapt to the generation of continuous, time series, and discrete data and maintain the original distribution of features and the correlation between them at a good level of privacy. Torkzadehmahani et al. [58] introduced a differential privacy condition GAN (CGAN) [59] training framework based on clipping and perturbation strategy, which generates synthetic data and corresponding labels while preserving the privacy of training datasets and uses RDP accountant to track the privacy budget of expenses. Liu et al. [60] proposed a GAN model for privacy protection, which achieves differential privacy by adding carefully designed noise to the clipping gradient in the process of model learning, uses the moment accountant strategy to improve the stability and compatibility of the model by controlling the loss of privacy, and generates high-quality synthetic data while retaining the required available data under a reasonable privacy budget. Ha and Dang [61] proposed a local differential privacy GAN model for noise data generation, which establishes a generative model by clipping the gradient in the model and adding Gaussian noise to the gradient to ensure the differential privacy. Chen et al. [62] proposed gradient-sanitized WGAN, which allows the publication of sanitized sensitive data under strict privacy guarantee and can more accurately distort gradient information so as to train deeper models and generate more information samples. Yang et al. [63] proposed a differential privacy gradient penalty WGAN (WGAN-GP) [64] to train a generative model with privacy protection function, which can provide strong privacy protection for sensitive data and generate high-quality synthetic data. Beaulieu-Jones et al. [65] used the auxiliary classifier GAN (AC-GAN) [66] with different privacy to generate simulated synthetic participants very similar to Systolic Blood Pressure Trial participants, which can generate synthetic participants and promote secondary analysis and repeatability investigation of clinical datasets by strengthening data sharing and protecting participants’ privacy. Fan and Pokkunuru [67] proposed a differential privacy solution for generating high-quality synthetic network flow data, which uses new clipping bound decay and privacy model selection to improve the quality of synthetic data and protects the privacy of sensitive training data by training GAN model with differential privacy. Zhang et al. [68] proposed a privacy publishing model based on GAN for graphs (NetGAN) [69], which can maintain high data utility in degree distribution and satisfy (ε,δ)-differential privacy.

Data perturbation can achieve privacy preserving by adding differential privacy noise to training data when using GAN generated synthetic data. Li et al. [70] proposed a graph data privacy protection method using GAN to perform an anonymization operation on graph data, which makes it possible to fully learn the characteristics of graph without specifying specific features and ensures the privacy performance of anonymous graph by adding differential privacy noise to the probability adjacency matrix in the process of graph generation. Neunhoeffer et al. [71] proposed differential privacy post-GAN boosting, which combines the samples produced by the generator sequence obtained during GAN training to create a high-quality synthetic dataset and reweights the generated samples using the private multiplication weight method [72]. Indhumathi and Devi [73] proposed healthcare Cramér GAN, which only adds differential privacy noise to the identified quasi identifiers, and the final result is combined with sensitive attributes, where the anonymous medical data are used as the real data for training Cramér GAN, Cramér distance is used to improve the efficiency of the model, and the synthetic data generation by health care GAN can provide high privacy and overcome various attacks. Imtiaz et al. [74] proposed a GAN combined with differential privacy mechanism to generate a real privacy smart health care dataset by directly adding noise to the aggregated data record, which can generate high-quality synthetic and differential privacy datasets and retain the statistical characteristics of the original dataset.

By using label perturbation of differential privacy noise, Papernot et al. [78] constructed the private aggregation of teacher ensembles (PATE), which provides a strong privacy guarantee for training data. The mechanism combines multiple models trained by disjoint datasets in a black box way. Because these models rely directly on sensitive data, they are not published but used as “teacher” of the “student” model. Because Laplace noise will only add the output of teachers, the students can learn to predict the output chosen by Laplace noisy voting among all teachers and cannot directly access a single teacher, basic data, or parameters. PATE uses moment accountant to better track privacy costs. Building on the GAN and PATE frameworks, Jordon et al. [75] replaced the GAN discriminator with the PATE mechanism. Therefore, the discriminator satisfies differential privacy, needing a differentiated student version to allow back propagation to the generator. However, this mechanism requires the use of public data.

In objective function perturbation, existing work injects Laplace noise into the coefficients to construct differentially private loss function in GAN training. Zhang et al. [76] proposed a new privacy protection GAN, which perturbs the coefficients of the objective function by injecting Laplace noise into the latent space based on the function mechanism to ensure the differential privacy of the training data, and it is reliable to generate high-quality real synthetic data samples without divulging the sensitive information in the training dataset.

In addition, the current research mainly focuses on publishing privacy-preserving data in a statistical way rather than considering the dynamics and correlation of the context. Thus, on the basis of triple-GAN [79], Ho et al. [77] proposed a generative adversarial game framework with three players based on triple-GAN, which designed a new perceptron, namely differential privacy identifier, to enhance synthetic data in the way of differential privacy. This deep generative model can generate synthetic data while fulfilling the differential privacy constraint.

### 8.2. Differential Privacy Synthetic Data Generation with Federated Learning

In order to achieve distributed collaborative data analysis, collecting large-scale data is an important task. However, due to the privacy of sensitive data, it is difficult to collect enough samples. Therefore, using GAN can generate synthetic data that can be shared for data analysis. However, in the distributed setting, training GAN faces new challenges of data privacy. Therefore, the existing work provides a solution for differential privacy synthetic data collection by combining GAN and federated learning in a distributed setting. According to the FedAvg training algorithm of model aggregation and averaging, federated learning is achieved by coordinating distributed data with independent and identically distributed and non-IID to perform collaborative learning [80].

Similar to the idea of gradient perturbation, using weight perturbation can achieve differential privacy of a generative model by clipping weight and adding noise to weight in GAN training with federated learning. Machine learning modeler workflow relies on data checking, so it is excluded when direct checking is impossible in the private and decentralized data paradigm. In order to overcome this limitation, Augenstein et al. [81] proposed a differential privacy algorithm, which synthesizes examples representing private data by adding Gaussian noise to the weighted average update.

Gradient perturbation can also be used to ensure the privacy protection of training data in GAN training with federated learning. Chen et al. [62] extended the gradient-sanitized WGAN to train GAN with differential privacy in federated setting and remarked some subtle differences between their method and the method of [81]. Different hospitals jointly train the model through data sharing to diagnose COVID-19 pneumonia, which will also lead to privacy disclosure. In order to solve this problem, Zhang et al. [82] proposed a federated differential privacy GAN for detecting COVID-19 pneumonia, which can effectively diagnose COVID-19 without compromising the privacy under IID and non-IID settings. The distributed storage of data and the fact that data cannot be shared due to privacy reasons for the federal learning environment bringing new challenges to training GAN. Thus, Nguyen et al. [83] proposed a new federated learning scheme to generate realistic COVID-19 images for facilitating enhanced COVID-19 detection with GAN in edge cloud computing, and this scheme integrates a differential privacy solution at each hospital institution to enhance the privacy in federated COVID-19 data analytics. By adding Gaussian noise to the gradient update process of the discriminator, Xin et al. [84] proposed a differential privacy GAN based on federated learning by strategically combining Lipschitz condition and differential privacy sensitivity, which uses a serialized model-training paradigm to significantly reduce the communication cost. Considering that distributed data are often non-IID in reality, which brings challenges to modeling, Xin et al. further proposed universal private FL-GAN to solve this problem. These algorithms can provide strict privacy guarantee using different privacy, but they can also generate satisfactory data and protect the privacy of training data, even if the data is non-IID.

Furthermore, considering differential average-case privacy [18] enhancing privacy protection of federated learning, Triastcyn and Faltings [85] proposed a privacy protection data publishing framework using GAN in the federated learning environment for which the generator component is trained by the FedAvg algorithm to draw private artificial data samples and empirically evaluate the risk of information disclosure. It can generate high-quality labeled data to successfully train and verify the supervision model, significantly reducing the vulnerability of such models to model inversion attacks.

## 9. Open Problems

We survey that the current work focuses on the definitions, privacy-utility metrics, properties, and achieving mechanisms of GDP and LDP based on the information-theoretic channel model. Mir [14] obtained the exponential mechanism achieving GDP by minimizing mutual information on the expected distortion constraint. We can intuitively obtain binary randomized response mechanism, quaternary randomized response mechanism, and multivariate randomized response mechanism under the binary symmetric channel, quasi-symmetric channel, and strongly symmetric channel, respectively, in terms of the Equation (5) of the LDP definition. Wang et al. [38] obtained the *k*-subset mechanism by maximizing mutual information about LDP constraint. Although GDP and LDP have been studied based on the information-theoretic channel model, there are some open problems for different application scenarios and data types from the perspective of different types of information-theoretic channel in Table 17.

(1) New LDP from the perspective of information-theoretic channel. Because local users have different privacy preferences, Yang et al. [86] proposed personalized LDP. However, it is necessary to study personalized LDP from the perspective of information-theoretic channel and propose the corresponding achieving mechanism. Although LDP does not require a trusted third party, it regards all local data equally sensitive, which causes excessive protection resulting in utility disaster [87]. Thus, it is necessary to study the utility-optimized mechanism for the setting where all users use the same random perturbation mechanism. In addition, since the differences between sensitive and non-sensitive data vary from user to user, it needs to propose a personalized utility-optimized mechanism of individual data achieving high utility while maintaining privacy preserving of sensitive data. Holohan et al. [42] proposed optimal mechanism satisfying (ε,δ)-LDP for randomized response. The optimal mechanism of the randomized response needs to be analyzed and obtained from the perspective of information-theoretic channel. Moreover, a new LDP mechanism needs to be analyzed by using the average error probability [20] as the utility metric under the rate-distortion framework of LDP.

(2) LDP from the perspective of discrete sequence information-theoretic channel. Collecting multiuser high-dimensional data can produce rich knowledge. However, this brings unprecedented privacy concerns to the participants [88,89]. In view of the privacy leakage risk of high-dimensional data aggregation, using the existing LDP mechanism brings poor data utility. Thus, it is necessary to study the optimal LDP mechanism of aggregating high-dimensional data from the perspective of discrete sequence information-theoretic channel. Furthermore, correlations exist between various attributes of high-dimensional data. If the correlation is not modeled, then the high-dimensional correlated data using LDP also leads to poor data utility [90,91]. By constructing the discrete sequence information-theoretic channel model of high-dimensional correlated data aggregation using LDP under joint probability or Markov chain, a LDP mechanism suitable for high-dimensional correlated data aggregation needs to be provided.

(3) GDP from the perspective of continuous information-theoretic channel. For GDP, there is no work to show the direct relationship between GDP mechanisms and single symbol continuous information-theoretic channel model, such as Laplace mechanism, discrete Laplace mechanism, and Gaussian mechanism. RDP is a general privacy definition, but existing work did not provide RDP mechanisms under continuous information-theoretic channel model. Thus, RDP mechanisms need to be studied from the perspective of continuous information-theoretic channel. The continuous releasing of correlated data and their statistics has the potential for significant social benefits. However, privacy concerns hinder the wider use of these continuous correlated data [92,93]. Therefore, the corresponding GDP mechanism from the perspective of continuous multi-symbol information-theoretic channel needs to be studied by combining the joint probability or Markov chain for continuous correlated data releasing with DP. However, it is common that the data curators have different privacy preferences with their data. Thus, personalized DP [94] needs to be studied based on continuous information-theoretic channel model. Existing GDP mechanisms ignore the characteristics of data and directly perturb the data or query results, which will inevitably lead to poor data utility. Therefore, it is necessary to study adaptive GDP depending on characteristics of data [95] from the perspective of continuous information-theoretic channel. Since users have different privacy demands, aggregate data analysis with DP also has poor data utility. Thus, adaptive personalized DP [96] also needs to be studied based on the type of query function, data distribution, and privacy settings from the perspective of continuous information-theoretic channel.

(4) GDP and LDP from the perspective of multiuser information-theoretic channel. A large amount of individual data have aggregated for computing various statistics, query responses, classifiers, and other functions. However, these processes will release sensitive information compromising individual privacy [97,98,99,100]. Thus, when considering the aggregation of multiuser data, the GDP and LDP mechanisms need to be studied from the multiple access channel. Data collection of GDP and LDP has been mostly studied for homogeneous and independently distributed data. In real-world applications, data have an inherent correlation which without harnessing will lead to poor data utility [101,102]. Thus, when the multiuser data are correlated, the GDP and LDP mechanisms need to be studied from the perspective of the multiuser channel with correlated sources. With the acceleration of digitization, more and more high-dimensional data are collected and used for different purposes. When these distributed data are aggregated, they can become valuable resources to support better decision making or provide high-quality services. However, because the data held by each party may contain highly sensitive information, simply integrating local data and sharing the aggregation results will pose a serious threat to individual privacy [103,104]. Therefore, GDP and LDP mechanisms need to be studied from the perspective of the broadcast channel for data releasing and sharing of multi-party data.

(5) Adaptive differential privacy with GAN. Existing work can generate differential privacy synthetic data using GAN. However, because of the differential privacy noise introduced in the training, the convergence of GAN becomes even more difficult and leads to the poor utility of output generator at the end of training. Therefore, it is necessary to explore adaptive differential privacy synthetic data using GAN to generate high-quality synthetic data according to the real data distribution. Combining differential privacy definition and information-theoretic metrics, a new differential privacy loss function model of GAN needed to be studied, and the differential privacy loss function model meets the convergence and reaches the optimal solution. Based on differential privacy loss function model, it is needed to construct adaptive differential privacy model. Using GAN and its variants generates synthetic data under adaptive differential privacy model. To improve the quality of the synthetic data using adaptive differential privacy model, GAN modeling is achieved by more layers, more complex structures, or transfer learning. Moreover, speed of GAN training can be accelerated by reducing the privacy budget. To resolve mode collapse and non-convergence issues, it is necessary to conduct fine tuning of hyper parameters, such as learning rate and number of discriminator epochs. Furthermore, the proposed adaptive differential privacy model with GAN should be extended to a distributed setting by using federated learning, which explores data augmentation methods which can improve the non-IID problem.

## 10. Conclusions

This survey has compared and analyzed the GDP and LDP from the perspective of information-theoretic channel. We concluded that the one-try attack with prior knowledge brings privacy concerns under information-theoretic channel. We described and compared the information-theoretic channel models of GDP and LDP for different data types. We summarized and compared the information-theoretic definitions of GDP and LDP under their information-theoretic channel models and presented the unified information-theoretic definitions of GDP and LDP, respectively. We also made a comparative analysis between GDP (LDP) and other information-theoretic privacy definitions. We surveyed and compared the privacy-utility metrics, properties, and achieving mechanisms of GDP and LDP from the perspective of information-theoretic channel. Moreover, we reviewed the differential privacy synthetic data generation using GAN and GAN with federated learning, respectively. Considering the problem of privacy threat to different real-world applications of different data types, we discussed the open problems from the perspective of different types of information-theoretic channel. We want that the survey can serve as a tutorial for the reader grasping GDP and LDP based on the information-theoretic channel model, and our survey can provide a reference to the reader to conduct in-depth research on GDP and LDP based on different types of information-theoretic channel models.

## Figures and Tables

**Table 1:** Advantages and disadvantages of GDP and LDP.

Privacy Type	Advantage	Disadvantage
GDP	Better data utility	Needing trusted data collector
	Suitable for dataset of any scale	
LDP	Without needing trusted data collector	Poor data utility
		Not applicable to small scale dataset

**Table 2:** Common mathematical symbols.

Symbol	Description
*x*	Dataset
M	Randomized mechanism
ε	Privacy budget
δ	Probability without satisfying differential privacy
*X*	Input random variable of information-theoretic channel
*Y*	Output random variable of information-theoretic channel
p(y|x)	Channel transition probability matrix
p(x)	Probability distribution on source *X*
q(x)	Another probability distribution on source *X*
$D_{\alpha }\left(p\left(x\right)||q\left(x\right)\right)$	Rényi divergence
$H_{\alpha }\left(X\right)$	Rényi entropy
H(X)	Shannon entropy
$H_{\infty }\left(X\right)$	Min-entropy
$H_{\alpha }\left(X|Y\right)$	Conditional Rényi entropy
H(X|Y)	Conditional Shannon entropy
$H_{\infty }\left(X|Y\right)$	Conditional min-entropy
I(X;Y)	Mutual information
$I_{\infty }\left(X;Y\right)$	Max-information
$I_{\infty }^{\beta }\left(X;Y\right)$	β-approximate max-information
$D_{\mathrm{KL}}\left(p\left(x\right)||q\left(x\right)\right)$	Kullback–Leibler divergence
$\Delta_{f}\left(p\left(x\right),q\left(x\right)\right)$	*f*-divergence
$||p\left(x\right)-{q\left(x\right)||}_{TV}$	Total variation distance
$D_{\infty }\left(p\left(x\right)||q\left(x\right)\right)$	Max-divergence
$D_{\infty }^{\delta }\left(p\left(x\right)||q\left(x\right)\right)$	δ-approximate max-divergence
$\bar{D}$	Expected distortion
$d(x_{i},y_{j})$	Single symbol distortion
$p_{E}$	Error probability
H	A class of functions
Γ	A divergence
$D_{\Gamma }^{H}\left(p\left(x\right),q\left(x\right)\right)$	H-restricted Γ-divergence
$D_{f}^{H}\left(p\left(x\right),q\left(x\right)\right)$	H-restricted *f*-divergence
$D_{R,\alpha }^{H}\left(p\left(x\right),q\left(x\right)\right)$	H-restricted Rényi divergence

**Table 3:** Information-theoretic channel models of GDP and LDP.

Privacy Type	Data Type	Input		GDP and LDP Mapping	Real Output	Random Output	Adjacent Relationship
GDP [7]	Numerical data	Dataset	*X*	$\left\{p\left(z|x\right):p\left(z|x\right)\leq e^{\varepsilon }p\left(z|x^{\prime }\right)\right\}$	*Y*	*Z*	*x* and $x^{\prime }$ are adjacent datasets.
LDP	Categorical data	Data item	*X*	$\left\{p\left(z|x\right):p\left(z|x\right)\leq e^{\varepsilon }p\left(z|x^{\prime }\right)\right\}$	*X*	*Z*	*x* and $x^{\prime }$ are different.

**Table 4:** GDP definitions using different information-theoretic metrics.

Existing Work	Privacy Type	Information-Theoretic Metric	Formula	Description
DP [7]	ε-DP	Channel transition probability	$p\left(z|x\right)\leq e^{\varepsilon }p\left(z|x^{\prime }\right)$	The transition probability matrix is used as the GDP mapping.
DP [8]	(ε,δ)-DP	*f*-divergence	$\Delta_{e^{\varepsilon }}=\max d_{e^{\varepsilon }}\left(p\left(z|x\right),p\left(z|x^{\prime }\right)\right)$ $d_{e^{\varepsilon }}=\max\left\{p\left(z|x\right)-e^{\varepsilon }p\left(z|x^{\prime }\right),p\left(z|x^{\prime }\right)-e^{\varepsilon }p\left(z|x\right),0\right\}$ $\Delta_{e^{\varepsilon }}\left(p\left(z|x\right),p\left(z|x^{\prime }\right)\right)\leq \delta$	*f*-divergence includes KL-divergence.
DP [9]	ε-DP	Max-divergence	$D_{\infty }(p\left(z|x\right)||p\left(z|x^{\prime }\right))\leq \varepsilon$ $D_{\infty }(p\left(z|x^{\prime }\right)||p\left(z|x\right))\leq \varepsilon$	Since the max-divergence is not symmetric and does not satisfy triangular inequality, the reciprocal of equation must be true.
	(ε,δ)-DP		$D_{\infty }^{\delta }(p\left(z|x\right)||p\left(z|x^{\prime }\right))\leq \varepsilon$ $D_{\infty }^{\delta }(p\left(z|x^{\prime }\right)||p\left(z|x\right))\leq \varepsilon$	
(α,ε)-RDP [11]	ε-DP	Rényi divergence	$D_{\alpha }(p\left(z|x\right)||p\left(z|x^{\prime }\right))\leq \varepsilon$	When α→∞, (α,ε)-RDP is ε-DP according to max-divergence. If M is ε-DP, then M is $\left(\alpha ,\frac{1}{2}\varepsilon^{2}\alpha \right)$-RDP [23].
	$\left(\varepsilon +\frac{\log\frac{1}{\delta }}{\alpha -1},\delta \right)$-DP			If M is (α,ε)-RDP, then it also satisfies $\left(\varepsilon +\frac{\log\frac{1}{\delta }}{\alpha -1},\delta \right)$-DP.
Capacity bounded DP [25]	(ε,δ)-DP	H-restricted divergence	$D_{\Gamma }^{H}\left(p\left(z|x\right),p\left(z|x^{\prime }\right)\right)\leq \varepsilon$	An adversary cannot distinguish between p(z|x) and $p(z|x^{\prime })$ beyond ε in the function class H, where Γ is the *f*-divergence.
	(α,ε)-RDP			An adversary cannot distinguish between p(z|x) and $p(z|x^{\prime })$ beyond ε in the function class H, where Γ is the Rényi divergence.

**Table 5:** Comparative analysis of GDP and other information-theoretic privacy definitions.

Existing Work	Information-Theoretic Privacy Definition	Formula	Description	Relationship to GDP	Stronger or Weaker than GDP
[26]	ε-information privacy	$e^{-\varepsilon }\leq \frac{p(x|z)}{p(x)}\leq e^{\varepsilon }$	When the output is given, the posterior and prior probabilities of the input *x* do not change significantly.	ε-information privacy ⇒2ε-DP	ε-information privacy is stronger than 2ε-DP.
[27]	ε-strong DP	$\sup_{z,x,x^{\prime }}\frac{p(z|x)}{p(z|x^{\prime })}\leq e^{\varepsilon },\forall z,x,x^{\prime }$	ε-strong DP relaxes the adjacent datasets assumption.	ε-strong DP ⇒ε-information privacy ε-information privacy ⇒2ε-strong DP ε-information privacy ⇒$\frac{\varepsilon }{H(X)}$-worst-case divergence privacy $\frac{\varepsilon }{H(X)}$-worst-case divergence privacy ⇒$\frac{\varepsilon }{H(X)}$-divergence privacy ε-DP ⇒(ε,δ)-DP	ε-strong DP is stronger than ε-information privacy. ε-information privacy is stronger than 2ε-DP. ε-DP is stronger than (ε,δ)-DP.
	ε-information privacy	The same as above.	The same as above.		
	Worst-case divergence privacy	$H\left(S\right)-\min_{z}H\left(S|Z=z\right)=\varepsilon H\left(S\right)$	Some private data *S* are correlated with some non-private data *X*.		
[12]	ε-identifiability	$p\left(x|z\right)\leq e^{\varepsilon }p\left(x^{\prime }|z\right)$	Two adjacent datasets cannot be distinguished from the posterior probabilities after observing the output dataset, which makes any individual’s data hard to identify.	ε-identifiability ⇒$\left[\varepsilon -\max\ln\frac{p(x)}{p(x^{\prime })},\varepsilon \right]$-DP ε-DP ⇒$\left[\varepsilon ,\varepsilon +2\max\ln\frac{p(x)}{p(x^{\prime })}\right]$-mutual-information privacy	ε-identifiability is stronger than $\left[\varepsilon -\max\ln\frac{p(x)}{p(x^{\prime })},\varepsilon \right]$-DP. ε-DP is stronger than $\left[\varepsilon ,\varepsilon +2\max\ln\frac{p(x)}{p(x^{\prime })}\right]$-mutual-information privacy.
	ε-mutual-information privacy	I(X;Z)≤ε	Mutual-information privacy measures the average amount of information about *X* contained in *Z*.		
[13]	ε-mutual-information DP	$\sup_{p(x_{i})}I\left(x_{i};Z|X^{-i}\right)\leq \varepsilon$	The same as ε-mutual-information privacy in work [12] above, and $X^{-i}$ represents the input dataset except the *i*-th element.	ε-DP⇒ε-mutual information DP ⇒(ε,δ)-DP	ε-DP is stronger than ε-mutual-information DP. ε-mutual-information DP is stronger than (ε,δ)-DP.

**Table 6:** Comparative analysis of LDP and other information-theoretic privacy definitions.

Existing Work	Information-Theoretic Privacy Definition	Formula	Description	Relationship to LDP	Stronger or Weaker than LDP
[12,19]	ε-mutual-information privacy	The same as Table 5 above.	The same as Table 5 above.	ε-local information privacy ⇒ε-mutual-information privacy ε-local information privacy ⇒2ε-LDP ε-LDP ⇒ε-local information privacy	ε-local information privacy is stronger than 2ε-LDP. ε-LDP is stronger than ε-local information privacy.
[19,26]	ε-local information privacy				
[21,26]	ε-local information privacy	The same as Table 5 above.	The same as Table 5 above.	ε-LDP ⇒ε-local information privacy ε-local information privacy ⇒2ε-LDP ε-SRLIP ⇒ε-local information privacy	ε-LDP is stronger than ε-local information privacy. ε-local information privacy is stronger than 2ε-LDP.
[21]	ε-SRLIP	$e^{-\varepsilon }\leq \frac{p(z|s,x_{1},\ldots ,x_{m})}{p(z|x_{1},\ldots ,x_{m})}\leq e^{\varepsilon }$	SRLIP satisfies ε-LIP for the attacker accessing some data $\left\{x_{1},\ldots ,x_{m}\right\}$ of a user and does not leak sensitive data *s* beyond the knowledge the attacker gained from the side channel.		

**Table 7:** Privacy metrics of GDP under information-theoretic channel model.

Existing Work	Privacy Metric	Formula	Description	Bound
[16]	Maximal leakage	$ML\left(p\left(z|x\right)\right)=\max_{p(x)}\left(H_{\infty }\left(X\right)-H_{\infty }\left(X|Z\right)\right)$	The maximal leakage of channel p(z|x) is the maximal reduction in uncertainty of *X* when *Z* is given, which is taken by maximizing over all input distributions of the attacker’s side information.	$ML\left(p\left(z|x\right)\right)\leq d\varepsilon \log_{2}e+\log_{2}m$ with spheres $\left\{x\in {\left\{0,1\right\}}^{n}|d\left(x,x_{i}\right)\leq d\right\}$ of radius *d* and center $x_{i}$.
[7]	Min-entropy leakage	$I_{\infty }\left(X;Z\right)=H_{\infty }\left(X\right)-H_{\infty }\left(X|Z\right)$	The min-entropy leakage corresponds to the ratio between the probabilities of attack success with a priori and a posterior.	$I_{\infty }\left(X;Z\right)\leq n\log_{2}\frac{\upsilon e^{\varepsilon }}{\upsilon -1+e^{\varepsilon }}$ with υ≥2
	Worst-case leakage	$C_{\infty }=\max_{p(x)}I_{\infty }\left(X;Z\right)$	The same as maximal leakage above.	
[29]	Mutual information	I(X;Z)	The mutual information denotes the amount of information leaked on *X* given *Z*.	I(X;Z)≤3εnI(X;Z)≥n(1−2η) with $\delta \geq 2^{-C(\varepsilon ,\eta )n}$, 0<ϵ,η<1, and a constant C(ε,η)>0
[30]	Min-entropy leakage	The same as above.	The same as above.	$I_{\infty }\left(X;Z\right)\leq \log\left(\sum_{t=1}^{q}\exp\left(\varepsilon d_{t}\right)\right)$, where *q* is the number of connected components of induced adjacency graph, and $d_{t}$ is the diameter of the *t*-th connected component.
[14]	Mutual information	I(X;Z)	The same as above.	–
[13]	α-mutual-information	$I_{\alpha }\left(X;Z\right)=\min_{p(z)}D_{\alpha }\left(p\left(z|x\right)||p\left(z\right)p\left(x\right)\right)$	The notion of α-mutual-information is the generalization of mutual information using Rényi information measures.	$\sup_{p(x_{i})}I_{\alpha }\left(x_{i};Z|X^{-i}\right)\leq \varepsilon$
[31]	Max-information	$I_{\infty }\left(X;Z\right)=\log_{2}\sup_{x,z\in (X,Z)}\frac{p(x,z)}{p(x)p(z)}$	Maximum information is a correlation measure, similar to mutual information, which allows to bound the change of the conditional probability of an event relative to prior probability.	$I_{\infty }\left(X;Z\right)\leq \log_{2}e\cdot \varepsilon n$ and $I_{\infty }^{\beta }\left(X;Z\right)\leq \log_{2}e\cdot \left(\varepsilon^{e}\frac{n}{2}+\varepsilon \sqrt{\frac{n\ln\frac{2}{\beta }}{2}}\right)$ for ε-DP $I_{\infty }^{\beta }\left(X;Z\right)=O\left(n\varepsilon^{2}+n\sqrt{\frac{\delta }{\varepsilon }}\right)$ for (ε,δ)-DP
	β-approximate max-information	$I_{\infty }^{\beta }\left(X;Z\right)=\log_{2}\sup_{O\subseteq (X\times Z),p((x,z)\in O)>\beta }\frac{p((x,z)\in O)-\beta }{p(x)p(z)}$		
[11]	Rényi divergence	$D_{\alpha }(p\left(z|x\right)||p\left(z|x^{\prime }\right))$	A natural relaxation of GDP based on the Rényi divergence.	–
[25]	H-restricted divergences	$D_{\Gamma }^{H}\left(p\left(z|x\right),p\left(z|x^{\prime }\right)\right)$	The privacy loss is measured in terms of a divergence Γ between output distributions of a mechanism on datasets that differ by a single record restricted to functions in H.	$D_{\mathrm{KL}}^{H}\left(p\left(z|x\right),p\left(z|x^{\prime }\right)\right)\leq 8\sqrt{\varepsilon }$
[32,33]	Privacy budget	$\varepsilon =\ln\frac{p(z|x)}{p(z|x^{\prime })}$	The privacy budget represents the level of privacy preserving.	–

**Table 8:** Utility metrics of GDP under information-theoretic channel model.

Existing Work	Utility Metric	Formula	Description	Bound
[7]	Expected distortion	$U\left(Y,Z\right)=\sum_{y}\sum_{z}p\left(y\right)p\left(z|y\right)d\left(y,z\right)$	How much information about the real answer can be obtained from the reported answer to average.	$U\left(Y,Z\right)\leq \frac{e^{\varepsilon n}\left(1-e^{\varepsilon }\right)}{e^{\varepsilon n}\left(1-e^{\varepsilon }\right)+c\left(1-e^{\varepsilon n}\right)}$ with |{z|d(y,z)=d}|=c
[14]	Expected distortion	$\sum_{x}\sum_{z}p\left(x\right)p\left(z|x\right)d\left(x,z\right)$	The same as above.	–
[32]	Fidelity	$\left|\right|\cdot {\left|\right|}_{1}$	The fidelity of a pair of transition probability distributions is $L_{1}$-distortion metric.	–
[33]	Mutual information	I(X;Z)	Mutual information captures the amount of information shared by two variables, that is to say, quantifying how much information can be preserved when releasing a private view of the data.	–

**Table 9:** Privacy metrics of LDP under information-theoretic channel model.

Existing Work	Privacy Metric	Formula	Description	Bound
[17]	KL-divergence	$D_{\mathrm{KL}}(p\left(z|x\right)||p\left(z|x^{\prime }\right))$	The general result bounds the KL-divergence between distributions p(z|x) and $p(z|x^{\prime })$ by the privacy budget ε and the total variation distance between p(x) and q(x) of the initial distributions of the *X*.	$D_{\mathrm{KL}}(p\left(z|x\right)||p\left(z|x^{\prime }\right))+D_{\mathrm{KL}}(p\left(z|x^{\prime }\right)||p\left(z|x\right))\leq 4{\left(e^{\varepsilon }-1\right)}^{2}||p\left(x\right)-q\left(x\right){\left|\right|}_{TV}^{2}$
[34,35]	Mutual information	I(X;Z)	The same as Table 7 above.	–
[4,5,37,38]	Privacy budget	$\varepsilon =\ln\frac{p(z|x)}{p(z|x^{\prime })}$	The same as Table 7 above.	–
Average privacy [39]	Conditional entropy	$\frac{H(X|Z,P)}{H(X|P)}$	Privacy metric is the fraction of sensitive information that is retained from the aggregator with prior knowledge *P*.	–

**Table 10:** Utility metrics of LDP under information-theoretic channel model.

Existing Work	Utility Metric	Formula	Description	Bound
[34,35,37]	Expected Hamming distortion	$E\left[d\left(x,z\right)\right]=p\left(x\neq z\right)=\sum_{x}\sum_{z}p\left(x\right)p\left(z\neq x|x\right)$	Hamming distortion measures the utility of a channel p(z|x) in terms of the worst-case Hamming distortion over source distribution p(x).	–
[5]	*f*-divergence	$D_{f}(p\left(z|x\right)||p\left(z|x^{\prime }\right))=\sum_{x}p\left(z|x^{\prime }\right)f\left(\frac{p(z|x)}{p(z|x^{\prime })}\right)$	*f*-divergence measures statistical discrimination between distributions p(z|x) and $p(z|x^{\prime })$ by the privacy budget ε and the total variation distance between p(x) and q(x) of the initial distributions of the *X*.	$D_{\mathrm{KL}}(p\left(z|x\right)||p\left(z|x^{\prime }\right))+D_{\mathrm{KL}}(p\left(z|x^{\prime }\right)||p\left(z|x\right))\leq \frac{2\left(1+\delta \right){\left(e^{\varepsilon }-1\right)}^{2}}{e^{\varepsilon }+1}||p\left(x\right)-q\left(x\right){\left|\right|}_{TV}^{2}$
	Mutual information	I(X;Z)	The same as Table 8 above.	$I\left(X;Y\right)\leq \frac{1}{2}\left(1+\delta \right)P\left(T\right)P\left(T^{c}\right)\varepsilon^{2}$ with $T\in \arg\min_{A\subseteq X}\left|P\left(A\right)-\frac{1}{2}\right|$ for a given distribution *P* and partitioning *X* into two parties *T* and $T^{c}$
[36]	Expected distortion	$\sum_{x}\sum_{z}p\left(x\right)p\left(z|x\right)d\left(x,z\right)$	A channel p(z|x) yields a small distortion between input and output sequences with respect to a given distortion measure.	–
Average error probability [20]	Expected Hamming distortion	$p_{E}=\sum_{x}p\left(x\right)\sum_{x\neq z}p\left(z|x\right)$	The average error probability is defined to be the expected Hamming distortion between the input and output data based on maximum a posterior estimation.	$p_{E}=\frac{n-1}{n-1+e^{\varepsilon }}$
[38]	Mutual information	I(X;Z)	The same as Table 8 above.	$\sup_{p(z|x)}I\left(X;Z\right)=\max_{k=\lfloor \beta \rfloor }^{\left\lceil \beta \right\rceil }\{\frac{k\cdot e^{\varepsilon }\log\frac{m\cdot e^{\varepsilon }}{k\cdot e^{\varepsilon }+m-k}\log\frac{m}{k\cdot e^{\varepsilon }+m-k}}{k\cdot e^{\varepsilon }+m-k}$} with $\beta =\frac{(\varepsilon e^{\varepsilon }-e^{\varepsilon }+1)m}{{\left(e^{\varepsilon }-1\right)}^{2}}$
Distribution utility [39]	Mutual information	$\frac{I(Z;P)}{I(X;P)}$	Utility metric is the fraction of relevant information after accessing to prior knowledge *P* or tally vector $T={\left(T_{x}\right)}_{x\in X}$ and $T_{x}=\left|\left\{i:x_{i}=x\right\}\right|$.	–
Tally utility [39]	Entropy Mutual information	$\frac{I(Z;T)}{H(T)}$		

**Table 11:** Properties of GDP under information-theoretic channel model.

Existing Work	Privacy Type	Privacy Property	Information-Theoretic Metric	Formal Description
[16]	GDP	Sequential composition	Maximal leakage	$ML\left(C_{1}+C_{2}\right)\leq ML\left(C_{1}\right)+ML\left(C_{2}\right)$ for the sequential composition $C_{1}+C_{2}$ of channels $C_{1}$ and $C_{2}$. When $C_{1}$ is $\varepsilon_{1}$-DP and $C_{2}$ is $\varepsilon_{2}$-DP, $C_{1}+C_{2}$ is $\varepsilon_{1}+\varepsilon_{2}$-DP.
		Parallel composition		$ML\left(C_{1}\times C_{2}\right)=ML\left(C_{1}\right)+ML\left(C_{2}\right)$ for the parallel composition $C_{1}\times C_{2}$ of channels $C_{1}$ and $C_{2}$. When $C_{1}$ is $\varepsilon_{1}$-DP and $C_{2}$ is $\varepsilon_{2}$-DP, $C_{1}\times C_{2}$ is $\max\{\varepsilon_{1},\varepsilon_{2}\}$-DP.
[8]	GDP	Sequential composition	*f*-divergence	$\Delta_{\alpha \alpha^{\prime }}\left(p\left(z|x\right),p\left(z|x^{\prime }\right)\right)\leq \Delta_{\alpha }\left(p\left(z|x\right),p\left(z|x^{\prime }\right)\right)+\max_{x}\Delta_{\alpha^{\prime }}\left(p\left(z|x\right),p\left(z|x^{\prime }\right)\right)$, where $\Delta_{\alpha }$, the same as Table 4 above.
[11]	RDP	Post-processing	Rényi divergence	If there is a randomized mapping $g:R\to R^{\prime }$, then $D_{\alpha }(p\left(z|x\right)||p\left(z|x^{\prime }\right))\geq D_{\alpha }(g\left(p\left(z|x\right)\right)||g\left(p\left(z|x^{\prime }\right)\right))$.
		Group privacy		If M:x→R is (α,ε)-RDP, $g:x^{\prime }\to x$ is $2^{c}$-stable and $\alpha \geq 2^{c+1}$, then M∘g is $\left(\frac{\alpha }{2^{c}},3^{c}\varepsilon \right)$-RDP.
		Sequential composition		If $M_{1}:x\to R_{1}$ is $\left(\alpha ,\varepsilon_{1}\right)$-RDP and $M_{2}:R_{1}\times x\to R_{2}$ is $\left(\alpha ,\varepsilon_{2}\right)$-RDP, then the mechanism $\left(M_{1},M_{2}\right)$ satisfies $\left(\alpha ,\varepsilon_{1}+\varepsilon_{2}\right)$-RDP.
[25]	Capacity bounded DP	Post-processing	H-restricted divergences	H, G, and I are function classes such that for any g∈G and i∈I, i∘g∈H. If mechanism M is (H,Γ)-capacity bounded DP with ε, then g∘M is also (I,Γ)-capacity bounded DP with ε for any g∈G.
		Convexity		$M_{1}$ and $M_{2}$ are two mechanisms which have the same range and provide (H,Γ)-capacity bounded DP with ε. If M is a mechanism which executes mechanism $M_{1}$ with probability π and $M_{2}$ with probability 1−π, then M is (H,Γ)-capacity bounded DP with ε.
		Sequential composition		H is the function class $H=\{H_{1}+H_{2}|h_{1}\in H_{1},h_{2}\in H_{2}\}$. If $M_{1}\left(x\right)$ and $M_{2}\left(x\right)$ are $\left(H_{1},\Gamma \right)$ and $\left(H_{2},\Gamma \right)$ capacity bounded DP with $\varepsilon_{1}$ and $\varepsilon_{2}$, respectively, then the combination $\left(M_{1},M_{2}\right)$ is (H,Γ) capacity bounded DP with $\varepsilon_{1}+\varepsilon_{2}$.
		Parallel composition		H is the function class $H=\{H_{1}+H_{2}|h_{1}\in H_{1},h_{2}\in H_{2}\}$. If $M_{1}\left(x_{1}\right)$ and $M_{2}\left(x_{2}\right)$ are $\left(H_{1},\Gamma \right)$ and $\left(H_{2},\Gamma \right)$ capacity bounded DP with $\varepsilon_{1}$ and $\varepsilon_{2}$ respectively, and the datasets $x_{1}$ and $x_{2}$ are disjoint, then the combination $\left(M_{1}\left(x_{1}\right),M_{2}\left(x_{2}\right)\right)$ is (H,Γ) capacity bounded DP with $\max\{\varepsilon_{1},\varepsilon_{2}\}$.
[3]	GDP LDP	Privacy-utility monotonicity	Mutual information	The mutual information decreases as the decreasing of the privacy budget, and vice versa

**Table 12:** GDP mechanisms under information-theoretic channel model.

Existing Work	Privacy Type	Model	Objective Function		Constraint Condition		Mechanism	Solution	Description
[7]	GDP	Maximal utility	Expected distortion	$U\left(Y,Z\right)=\sum_{y}\sum_{z}p\left(y\right)p\left(z|y\right)d\left(y,z\right)$	Min-entropy leakage	$I_{\infty }\left(X;Z\right)=H_{\infty }\left(X\right)-H_{\infty }\left(X|Z\right)$	$p\left(z|y\right)=\frac{\alpha }{{\left(e^{\varepsilon }\right)}^{d}}$, where d=d(y,z), $\alpha =\frac{{\left(e^{\varepsilon }\right)}^{n}\left(1-e^{\varepsilon }\right)}{{\left(e^{\varepsilon }\right)}^{n}\left(1-e^{\varepsilon }\right)+c\left(1-{\left(e^{\varepsilon }\right)}^{n}\right)}$, and *c* the same as Table 8 above.	Graph symmetry induced by the adjacent relationship of adjacent datasets.	Optimal randomization mechanism provides the better utility while guaranteeing ε-DP.
[14]	GDP	Risk-distortion	Mutual information	$\inf_{p(z|x)}I\left(X;Z\right)$	Expected distortion	∑p(x)∑p(z|x)d(x,z)≤D	$p\left(z|x\right)=\frac{p(z)\exp(-\varepsilon d(x,z))}{Z(x,\varepsilon )}$, where Z(x,ε) is a normalization function.	Lagrangian multipliers.	Conditional probability distribution is DP mapping, which minimizes the privacy risk given a distortion constraint.
[33]	GDP	Constrained maximization program	Mutual information	maxI(X;Z)	GDP	$\sup\frac{p(z|x)}{p(z|x^{\prime })}\leq \exp\left(\varepsilon \right)$	$$p\left(z|x\right)=\left\{\begin{array}{cc} p(z=x|x), & x=z\\ \frac{1-p(z=x|x)}{m-1}, & x\neq z \end{array}\right.$$	Definition of GDP.	When *x* is transformed into *z* and z=x, the conditional transition probability is p(z=x|x). When z≠x, the conditional transition probability is $\frac{1-p(z=x|x)}{m-1}$ under strongly symmetric channel.

**Table 13:** LDP mechanisms under information-theoretic channel model.

Existing Work	Privacy Type	Model	Objective Function		Constraint Condition		Mechanism		Solution	Description
[34,35,37]	LDP	Rate-distortion function	Mutual information	$\min_{p(z|x)}I\left(X;Z\right)$	Expected Hamming distortion	$\sum_{x}\sum_{z}p\left(x\right)p\left(z|x\right)d\left(x,z\right)\leq D$	Binary channel	$\varepsilon =\log\frac{1-D}{D}$	Memoryless symmetric channel.	LDP is just a function of the channel, and the worst-case Hamming distortion on source distribution p(x) measures the utility of a channel p(z|x).
							Discrete alphabet	$\varepsilon =\log\left(m-1\right)+\log\frac{1-D}{D}$		
[5]	LDP	Constraint maximization problem	KL-divergence Mutual information	$\max_{p(z|x)}D_{\mathrm{KL}}(p\left(z|x\right)||p\left(z|x^{\prime }\right))$ $\max_{p(z|x)}I\left(X;Z\right)$	LDP	$\varepsilon =\ln\frac{p(z|x)}{p(z|x^{\prime })}$	Binary randomized response	$$p\left(z|x\right)=\left\{\begin{array}{cc} \frac{e^{\varepsilon }}{1+e^{\varepsilon }}, & x=z\\ \frac{1}{1+e^{\varepsilon }}, & x\neq z \end{array}\right.$$	Solving the privacy-utility maximization problem is equivalent to solving finite-dimensional linear program.	The binary and multivariate randomized response mechanisms are universally optimal in the low and high privacy regimes and well approximate the intermediate regime. The quaternary randomized mechanism satisfies (ε,δ)-LDP.
							Multivariate randomized response	$$p\left(z|x\right)=\left\{\begin{array}{cc} \frac{e^{\varepsilon }}{n-1+e^{\varepsilon }}, & x=z\\ \frac{1}{n-1+e^{\varepsilon }}, & x\neq z \end{array}\right.$$		
							Quaternary randomized response	$$\begin{array}{cc} & \;0\;1\;\;2\;\;\;3\\ \begin{array}{c} 0\\ 1 \end{array} & \left(\begin{array}{cccc} \delta & 0 & \frac{1-\delta }{1+e^{\varepsilon }} & \frac{\left(1-\delta \right)e^{\varepsilon }}{1+e^{\varepsilon }}\\ 0 & \delta & \frac{\left(1-\delta \right)e^{\varepsilon }}{1+e^{\varepsilon }} & \frac{1-\delta }{1+e^{\varepsilon }} \end{array}\right) \end{array}$$		
[38]	LDP	Maximize utility	Mutual information	$\sup_{p(z|x)}I\left(X;Z\right)\leq I_{\beta }$ $I_{k}=\frac{k\cdot e^{\varepsilon }\log\frac{m\cdot e^{\varepsilon }}{k\cdot e^{\varepsilon }+m-k}\log\frac{m}{k\cdot e^{\varepsilon }+m-k}}{k\cdot e^{\varepsilon }+m-k}$ $\beta =\frac{(\varepsilon e^{\varepsilon }-e^{\varepsilon }+1)m}{{\left(e^{\varepsilon }-1\right)}^{2}}$	LDP	$\varepsilon =\ln\frac{p(z|x)}{p(z|x^{\prime })}$	*k*-subset mechanism	p(Z|x)=neεnk(keε+n−k),|Z|=k,x∈Znnk(keε+n−k),|Z|=k,x∉Z0,|Z|≠k	This problem maximizes mutual information when *x* is a sample according to the uniform distribution with probability $\frac{1}{n}$.	The mutual information bound is used as a universal statistical utility measurement, and the *k*-subset mechanism is the optimal ε-LDP mechanism.

**Table 14:** Membership inference attack and model extraction attack against GAN.

Existing Work	Attack Target	Attack Type	Attack Method	Characteristic	Attack Effect
[47]	Generative models	Membership inference	The discriminator can learn the statistical difference of distribution, detect overfitting and recognize the input as part of the training dataset.	The proposed attack has low running cost, does not need information about the attacked model, and has good generalization.	Defenses are either ineffective or lead to a significant decline in the performance of the generative models in terms of training stability or sample quality.
[48]	Generative models	Co-membership inference	The membership inference of the target data *x* is used as the optimization of the attacker’s network to search for potential codes to reproduce *x*, and the final reconstruction error is used to judge whether *x* is in the training data.	When the generative models are trained with large datasets, the co-membership inference attack is necessary to achieve success.	The performance of attacker’s network is better than that of previous membership attacks, and the power of co-membership attack is much greater than that of a single attack.
[49]	Generative models	Membership inference	The membership inference attack based on Monte Carlo integration only considers the small distance samples in the model.	This attack allows membership inference without assuming the type of generative models.	The success rate of this attack is better than that of previous studies on most datasets, and there are only very mild assumptions.
[50]	Generative models	Membership inference	This work proposed a general attack model based on reconstruction for which the model is suitable for all settings according to the attacker’s knowledge about the victim model.	This work provides a theoretically reliable attack calibration technology, which can continuously improve the attack performance in different attack settings, data modes, and training configurations in all cases.	This attack reveals the information of the training data used for the victim model.
[51]	GAN	Model extraction	This work studied the model extraction attack based on target and background knowledge from the perspectives of fidelity extraction and accuracy extraction.	Model extraction based on transfer learning can enable adversaries to improve the performance of their GAN model through transfer learning.	Attack model stealing the most advanced target model can be transferred to new fields to expand the application scope of extraction model.

**Table 15:** Differential privacy synthetic data generation with GAN.

Existing Work	GAN Type	Clipping Strategy	Perturbation Strategy	Privacy Loss Accountant
[55]	GAN	Clipping gradient	Gradient perturbation	Moment accountant
[56]	WGAN	Clipping weight	Gradient perturbation	Moment accountant
[45]	GAN	Clipping gradient	Gradient perturbation	Moment accountant
[58]	CGAN	Clipping gradient	Gradient perturbation	RDP accountant
[60]	GAN	Clipping gradient	Gradient perturbation	Moment accountant
[61]	GAN	Clipping gradient	Gradient perturbation	Moment accountant
[62]	WGAN	Clipping gradient	Gradient perturbation	RDP accountant
[63]	WGAN-GP	Clipping gradient	Gradient perturbation	Moment accountant
[65]	AC-GAN	Clipping gradient	Gradient perturbation	Moment accountant
[67]	GAN	Clipping gradient	Gradient perturbation	Moment accountant
[68]	NetGAN	Clipping gradient	Gradient perturbation	Privacy budget composition [9]
[70]	GAN	–	Data perturbation	–
[71]	GAN	–	Data perturbation	Advanced composition [9]
[73]	GAN	–	Data perturbation	–
[74]	GAN	–	Data perturbation	–
[75]	GAN	–	Label perturbation	Moment accountant
[76]	GAN	–	Objective function perturbation	Advanced composition
[77]	GAN	–	Differential privacy identifier	Privacy budget composition

**Table 16:** Differential privacy synthetic data generation with federated learning.

Existing Work	GAN Type	Clipping Strategy	Perturbation Strategy	Privacy Loss Accountant	Training Method
[81]	GAN	Clipping weight	Weight perturbation	RDP accountant	FedAvg algorithm
[62]	WGAN	Clipping gradient	Gradient perturbation	RDP accountant	FedAvg algorithm
[82]	GAN	Clipping weight	Gradient perturbation	Moment accountant	FedAvg algorithm
[83]	GAN	–	Gradient perturbation	–	FedAvg algorithm
[84]	GAN	Clipping gradient	Gradient perturbation	RDP accountant	Serial training
[85]	GAN	–	Differential average-case privacy	–	FedAvg algorithm

**Table 17:** Open problems of GDP and LDP from the perspective of different types of information-theoretic channel.

Scenario	Data Type	Privacy Type	Open Problem	Method	Information-Theoretic Foundation
Data collection	Categorical data	LDP	Personalized privacy demands	Rate-distortion framework	Discrete single symbol information-theoretic channel
			Poor data utility		
			Information-theoretic analysis of existing LDP mechanisms		
High-dimensional (correlated) data collection	Categorical data	LDP	Poor data utility	Rate-distortion framework Joint probability Markov chain	Discrete sequence information-theoretic channel
Continuous (correlated) data releasing	Numerical data	GDP	Information-theoretic analysis of existing GDP mechanisms	Rate-distortion framework Joint probability Markov chain	Continuous information-theoretic channel
			RDP mechanisms		
			Personalized privacy demands		
			Poor data utility		
Multiuser (correlated) data collection	Numerical data Categorical data	GDP LDP	Privacy leakage risk	Rate-distortion framework	Multiple access channel Multiuser channel with correlated sources
Multi-party data releasing					Broadcast channel
Synthetic data generation	Numerical data Categorical data	GDP LDP	Poor data utility	GAN GAN with federated learning	Information-theoretic metrics

## Data Availability

Not applicable.
